# Phase Ia Evaluation of LP-184 in Recurrent Glioblastoma: Safety, Pharmacokinetics, and Translational Optimization of CNS Exposure

**DOI:** 10.1158/2767-9764.CRC-26-0518

**Published:** 2026-10-27

**Authors:** Karisa C. Schreck, Bachchu Lal, Jianli Zhou, Hernando Lopez-Bertoni, Matthias Holdhoff, Reginald Ewesuedo, Kishor Bhatia, Marc Chamberlain, John Laterra

**Affiliations:** 1 Department of Neurology, Johns Hopkins University School of Medicine, Baltimore, Maryland.; 2 Sidney Kimmel Comprehensive Cancer Center, Johns Hopkins University School of Medicine, Baltimore, Maryland.; 3 Hugo W. Moser Research Institute at Kennedy Krieger, Baltimore, Maryland.; 4 Lantern Pharma Inc., Dallas, Texas.

## Abstract

**Purpose:**

Limited central nervous system (CNS) bioavailability and pharmacodynamics are obstacles to effective systemic therapies for glioblastoma (GBM). One strategy to overcome these challenges is drug combinations enhancing CNS penetration and/or tumor chemosensitivity. LP-184 (zirdafulven), a synthetic acylfulvene class alkylator, induces DNA damage and inhibits GBM cell viability in preclinical models. LP-184 is a prodrug converted to active metabolites by intracellular oxidoreductase prostaglandin reductase 1 that is overexpressed in >70% of GBM. DNA damage induced by LP-184 is O^6^-methylguanine-DNA methyltransferase (MGMT) agnostic and reversed by transcription-dependent transcription-coupled nucleotide excision repair (TC-NER).

**Patients and Methods:**

LP-184 was evaluated in a phase Ia study (NCT05933265) in 63 adult patients with advanced malignancies, including 16 patients with recurrent GBM. All patients with GBM received prior standard-of-care therapy, and most had received one or more additional therapies before enrollment.

**Results:**

Treatment was overall well tolerated. Patients with GBM experienced frequent grade 1 to 2 nausea and infrequent severe thrombocytopenia and transaminitis. Clinical pharmacokinetic analysis combined with published preclinical intratumoral bioavailability data (∼20% penetration) predicted that LP-184 at the recommended dose for expansion (RDE) would achieve cytotoxic levels if combined with spironolactone, a brain-penetrant ERCC3 degrader and TC-NER inhibitor that sensitizes GBM cells to LP-184 3- to 6-fold. We show that three daily doses of spironolactone deplete orthotopic GBM patient-derived xenograft (PDX) ERCC3 protein by ∼80% and increase tumor LP-184 cytotoxicity 2-fold.

**Conclusions:**

LP-184 is well tolerated at the RDE, and we establish a clinically translatable plan for dosing spironolactone in combination with LP-184 for a future phase Ib/IIa clinical trial.

**Significance:**

Treatment failure in GBM reflects inadequate drug brain exposure and DNA repair–mediated resistance. LP-184 (zirdafulven), a novel acylfulvene alkylator, generates MGMT-independent DNA lesions predominantly repaired by TC-NER. In a phase Ia dose-finding trial, LP-184 was well tolerated at the RDE in participants with recurrent GBM. Plasma drug levels achieved predicted effective systemic exposures but not brain concentrations based on projected 20% brain penetrance. Pharmacokinetic modeling indicates that NER inhibition could increase tumor chemosensitivity with the addition of spironolactone. The optimal dosing regimen for spironolactone combined with LP-184 was identified in orthotopic PDX models, facilitating advancement to phase Ib/IIa testing of LP-184 plus spironolactone.

**Trial Registration:**

ClinicalTrials.gov Identifier: NCT05933265.

## Introduction

Glioblastoma (GBM) is the most common malignant primary brain tumor in adults and remains uniformly fatal notwithstanding maximal safe resection and radiotherapy with concurrent and adjuvant temozolomide (TMZ), with a median overall survival of ∼15 to 18 months in protocol-eligible patients (1, 2). More than half of patients with newly diagnosed GBM do not benefit from first-line chemotherapy with TMZ due to the expression of O^6^-methylguanine-DNA methyltransferase (MGMT), which repairs TMZ-induced DNA damage (3). Effective systemic options are limited at recurrence, and resistance driven by diffuse infiltration and marked intratumoral heterogeneity contributes to the substantial unmet need for novel therapies (4–6). An additional obstacle is the blood–brain barrier (BBB), which restricts central nervous system (CNS) exposure for most systemically administered drugs (7–9). CNS therapeutics face unique challenges in achieving sufficient intratumoral concentrations and targeting both the enhancing and nonenhancing tumor compartments common in GBM (10).

LP-184, an alkylator of the acylfulvene class, requires conversion of the parent prodrug to an active metabolite by intracellular oxidoreductases, particularly prostaglandin reductase 1 (PTGR1; ref. 11). PTGR1 is overexpressed in many cancers relative to normal tissues, including GBM, providing selective cytotoxicity for LP-184 (12–14). Importantly, the alkylating effect of LP-184 and other acylfulvenes is independent of MGMT status, with DNA repair primarily occurring through the transcription-coupled nucleotide excision repair (TC-NER) or homologous recombination (HR) pathways (15–17). LP-184 demonstrates preclinical cytotoxicity as a single agent in multiple models, and antitumor activity is augmented in the setting of intrinsic and induced DNA repair deficiency and in combination with radiation (18–20).

Preclinical pharmacokinetic modeling and brain distribution studies further suggest that LP-184 crosses the BBB, with approximately 20% of systemic exposure reaching brain tumors and ∼10% reaching the contralateral brain (20). Antitumor efficacy and CNS delivery are supported by tumor volume reduction and survival prolongation in multiple orthotopic glioma models, including patient-derived mesenchymal glioblastoma xenografts (PDX), with good *in vivo* tolerability (20). Together, these data support the clinical evaluation of LP-184 for GBM, including the assessment of safety, tolerability, and preliminary antitumor activity, alongside correlative analyses to better define CNS penetration and pharmacodynamic evidence of DNA damage in tumor and/or surrogate compartments.

In parallel, compounds that sensitize cancer cells to LP-184 were actively explored, and spironolactone was identified as a likely candidate given its ability to inhibit HR and NER (21, 22). Spironolactone is a BBB-permeable aldosterone antagonist that inhibits DNA damage repair (DDR; specifically NER) by inducing proteasomal degradation of ERCC3 (XPB; refs. 23–25). Preclinical studies demonstrate that spironolactone sensitizes GBM models to LP-184 *in vitro* and *in vivo*, as evidenced by increased tumor cell DNA damage response, increased tumor cell killing, and increased GBM PDX response *in vivo* (20). By depleting ERCC3, spironolactone sensitizes GBM cells to LP-184, resulting in a 3- to 6-fold lower IC_50_ in multiple GBM cell models (26).

The current study evaluates LP-184 in a first-in-human phase Ia trial in adult patients with advanced solid tumors to determine the recommended dose for expansion (RDE; NCT05933265; ref. 27). Patients with recurrent GBM were enrolled in this study, and here, we report safety and tumor-specific outcomes in the GBM cohort. We also identify a clinically translatable plan for dosing the ERCC3 degrader and TC-NER inhibitor, spironolactone, in combination with LP-184 in preparation for a phase Ib/IIa study in patients with GBM (NCT07431216).

## Patients and Methods

### Study design and treatment

NCT05933265 is a phase Ia, first-in-human, dose escalation study evaluating the safety, tolerability, pharmacokinetics (PK), and preliminary antitumor activities of LP-184 in adult patients (≥18 years old) with advanced solid tumors, including GBM. Key inclusion criteria included relapsed or refractory disease, Karnofsky performance status >60, adequate organ function, measurable disease by RANO v2.0 (in patients with GBM), and life expectancy >3 months. In patients with GBM, a chronic stable dose of dexamethasone was required, as was prior initial treatment with surgery, TMZ-based chemoradiotherapy, and adjuvant TMZ (Supplementary Table S1). There were no limits on the number of prior treatments.

Dose escalation followed a Bayesian optimal interval (BOIN) design with a starting dose level of 0.01 mg/kg. Dose-limiting toxicities (DLT) were assessed during cycle 1. Patients received LP-184 intravenously over 30 (±5) minutes on days 1 and 8 of each 21-day cycle. Treatment continued until disease progression, unacceptable adverse events (AE), withdrawal of consent, loss to follow-up, protocol deviation, or at the discretion of the investigators. The study was conducted in accordance with the ethical standards in the Declaration of Helsinki and applicable Good Clinical Practice guidelines. The study protocol was approved by the appropriate institutional and/or national research ethics committee. Written informed consent was obtained from all patients or their legal representatives before enrollment.

### Endpoints and assessments

The primary objectives were to evaluate the safety and tolerability of LP-184 and determine the maximally tolerated dose (MTD) and RDE. Safety evaluations included clinical laboratory assessments, electrocardiograms, physical examinations, vital signs, and AE monitoring. AEs were graded according to the NCI Common Terminology Criteria for Adverse Events (CTCAE) v5.0. The MTD was defined as the highest dose level at which the isotonic estimate of the DLT rate is closest to the target toxicity rate of 0.3. The RDE was determined based on the totality of clinical data, including PK, safety, and efficacy.

Secondary endpoints include PK and preliminary antitumor activities (i.e., objective response rate, duration of response, and progression-free survival). Additionally, archival tumor samples were analyzed for PGTR1 expression when available; however, mandatory tissue submission was not required per protocol. Blood samples for pharmacokinetic analysis were collected at prespecified time points before the first infusion and at multiple timepoints over 24 hours after the first infusion. A PK trough sample was taken on cycle 1 day 8. Plasma concentration of LP-184 was measured using a validated LC/MS-MS assay. Tumor assessment was performed at baseline, after every two cycles during the first four cycles, and every four cycles thereafter. Response was evaluated using RECIST v1.1 (non-GBM patients) or RANO v2.0 (patients with GBM).

### PTGR1 quantification

Total RNA was extracted from sections of formalin-fixed, paraffin-embedded tissue blocks. Complementary DNA (cDNA) was synthesized from up to 2,000 ng of RNA using the High-Capacity cDNA Reverse Transcription Kit (Thermo Fisher Scientific, part no. 4368814) according to the manufacturer’s instructions. Reverse transcription–quantitative polymerase chain reaction (RT-qPCR) was performed in triplicate using TaqMan probe–based assays for the target gene *PTGR1* (Thermo Fisher Scientific, part no. 4331182, assay ID Hs00400932_m1) and two housekeeping genes, *ACTB* (assay ID Hs01060665_g1) and *GAPDH* (assay ID Hs03929097_g1), with reactions prepared using a commercial master mix (Thermo Fisher Scientific, part no. 4369016) and run on a ViiA7 Real-Time PCR System under standard cycling conditions. Cycle threshold (Ct) values were obtained, and relative gene expression was calculated using the comparative ΔCt method, normalizing the target gene to the mean of the two housekeeping genes. No-template controls, a positive control (cDNA from a xenograft with high *PTGR1* expression), and a negative control (cDNA from a xenograft with low or undetectable *PTGR1* expression) were included in each run.

For determination of the preliminary PTGR1 threshold, RT-qPCR was performed as above on the following 10 cell lines: K562 (ATCC, CCL-243), HCC-78 (ATCC, CRL-5872), NCI-H1666 (ATCC, CRL-5885), LNCaP (ATCC, CRL-1740), U-87 MG (ATCC, HTB-14), 8505C (ATCC, CRL-8305), MDA PCa 2b (ATCC, CRL-2422), LN-18 (ATCC, CRL-2610), NCI-H1944 (ATCC, CRL-5907), and NCI-H2228 (ATCC, CRL-5935).

### Preclinical studies

#### Tumor xenografts

The patient-derived GBM xenograft line Mayo39 was obtained from the Mayo Clinic (28) and cultured as previously described (26). It was tested for mycoplasma and short tandem repeat profiled prior to use, and low-passage cultures were used for *in vivo* experiments. For orthotopic xenografts, NOD-SCID mice received 10^5^ cells suspended in 2 μL phosphate-buffered saline (PBS) to the right caudate/putamen. To evaluate the kinetics of ERCC3 degradation, beginning on day 15 after implantation, mice received 1, 2, or 3 doses of spironolactone (35 mg/kg intraperitoneally, every 24 hours) or PBS control. Animals were sacrificed at the indicated times following spironolactone dosing, and tissues were isolated for immunoblot analysis. To determine how optimal spironolactone dosing affects tumor cell responses to LP-184, NOD-SCID mice were orthotopically implanted with Mayo39 cells as described above. On day 15 after implantation, mice began to receive three consecutive doses of spironolactone (35 mg/kg intraperitoneally, every 24 hours) or PBS as control ± LP-184 (4 mg/kg intravenously) administered 8 hours after the third dose of spironolactone. Animals were sacrificed 48 hours after LP-184 infusion, and tissues were processed for immunoblot.

#### Immunoblot analyses

Immunoblotting was performed as previously described (26) using NOVEX 4% to 12% Tris-glycine gradient gels (Thermo Fisher Scientific) and Amersham Protran nitrocellulose membranes (GE HealthCare). Membranes were probed with primary antibodies for ERCC3 (Aviva System Biology, ARP37963_P050), phosphorylated γH2AX (Cell Signaling Technology, #2577S for immunoblot), cleaved caspase-3 (Cell Signaling Technology, #9664 for immunoblot), or actin (Cell Signaling Technology, cat. #3700S) and secondary antibodies labeled with IRDye infrared dye (LI-COR Biosciences). Protein band intensities were quantified using the Odyssey Infrared Imager (LI-COR Biosciences) and analyzed using the Image Studio acquisition software from LI-COR imaging systems.

### Statistical analysis

Safety and efficacy analyses included all patients who received at least one dose of LP-184. Descriptive statistics were used to summarize safety events. The Fisher two-sided exact test (95% confidence interval) was used to compare the frequency of AEs between the GBM and non-GBM groups. Plasma concentration–time data from patients treated at dose 10 (*n* = 11) were analyzed using nonlinear regression with a one-phase exponential decay model to estimate the dynamics of LP-184. For preclinical studies, one- or two-way ANOVA and Dunnett or Tukey *post hoc* tests were used when comparing multiple variables. Data analysis and visualization were conducted using R (v4.1.0) and GraphPad Prism (v11.0.0).

## Results

### Patient characteristics and dosing

Sixteen patients with recurrent GBM enrolled in the phase Ia dose escalation study of LP-184 (overall, 63 patients were enrolled). See Table 1 for patient characteristics. Of these participants, all had GBM, isocitrate dehydrogenase (IDH) wild type, World Health Organization (WHO) grade 4. Eight of fourteen with known MGMT status had an unmethylated promoter (57%). Molecular tumor profiling was completed on pretreatment tissue from all tumors as clinically indicated (Supplementary Table S2). Nine of ten tested (90%) had a *TERT* promoter mutation, eight of twelve (67%) had *EGFR* amplification, five of six (83%) had homozygous deletion of *CDKN2A/CDKN2B*, five of twelve (42%) had the EGFRvIII translocation, and three of nine (33%) had *PTEN* single-nucleotide variants.

**Table 1.** Characteristics of patients with GBM.

Patients characteristics	Total (*N* = 16)
Age, years [median (range)]	56 (22–72)
Sex, *n* (%)	​
Male	10 (62%)
Female	6 (38%)
Race, *n* (%)	​
White	15 (94%)
Black	1 (6%)
ECOG performance status, *n*/*N* (%); determined in 5/16 patients	​
0	1/5 (20%)
1	4/5 (80%)
Karnofsky performance status, *n*/*N* (%); determined in 11/16 patients	​
70	3/11 (27%)
80	1/11 (9%)
90	7/11 (64%)
Prior systemic regimens, median (range)	2 (1–4)
TMZ, *n* (%)	16 (100%)
Lomustine, *n* (%)	3 (19%)
Concurrent TMZ during radiotherapy, *n* (%)	16 (100%)
Estimated cycles of TMZ maintenance after RT, median (range)	6 (0–15)
TMZ re-challenge, *n* (%)	2 (13%)
TMZ discontinuation due to AEs, *n* (%)	2 (13%)
Number of surgeries, *n* (%)	​
1	13 (71%)
2	3 (19%)
Number of radiation treatments, *n* (%)	​
1	13 (71%)
2	3 (19%)
Time from prior treatment	​
Months between completion of radiotherapy and start of LP-184, median (range)	11 (5–50)
Months between end of TMZ and start of LP-184, median (range)	5 (1–44)
IDH status, *n* (%)	​
Mutated	0
Wild type	16 (100%)
MGMT promoter methylation, *n*/*N* with reported status (%)	​
Methylated	6/14 (43%)
Unmethylated	8/14 (57%)
Dexamethasone dose at enrollment, *n* (%)	13 (81%)
1 mg daily	1 (6%)
2 mg daily	5 (31%)
4 mg daily	6 (38%)
8 mg daily	1 (6%)

The study population was heavily pretreated, with an average of two prior lines of therapy (range, 1–5). All participants received prior radiation and TMZ, with a median of six cycles of adjuvant TMZ therapy. Subsequent therapies received at recurrence included lomustine (19%), re-resection (19%), or re-radiation (19%) and re-challenge with TMZ (13%). The median time between prior adjuvant TMZ and initiating study treatment with LP-184 was 5 months (range, 1–44). The median time between completion of prior radiotherapy and initiating study treatment with LP-184 was 11 months (range, 5–50). Eighty-eight percent of patients were on a stable dose of dexamethasone at the time of study enrollment.

Patients with GBM were enrolled at dose level 4 (DL4) and all but one subsequent dose level (Supplementary Table S3). All patients with GBM have come off the study, with 14 (87.5%) patients having radiographic or clinical progression. Two patients discontinued treatment due to AEs, one (DL8) having prolonged thrombocytopenia and neutropenia leading to dose holds and the other patient (DL12) having transient transaminitis and an elevated total bilirubin meeting Hy’s law and the definition of a DLT. DL10 was established as the RDE based on the totality of safety and pharmacokinetic data.

### Toxicity

The range of treatment-related AEs in patients who received LP-184 on study with GBM (*n* = 16) was similar to the larger cohort (*n* = 47; ref. 27). No AEs were observed exclusively in patients with GBM (Fig. 1A). Nausea and/or vomiting was CTCAE grade 1 or 2 in all participants who experienced an AE. Of note, the study did not permit pretreatment with antiemetics during cycle 1 day 1; however, prophylactic antiemetics were permitted for subsequent treatments. In patients with alanine aminotransferase (ALT) elevation, one each of grade 3 and 4 AE was observed in patients with GBM.

**Figure 1.**
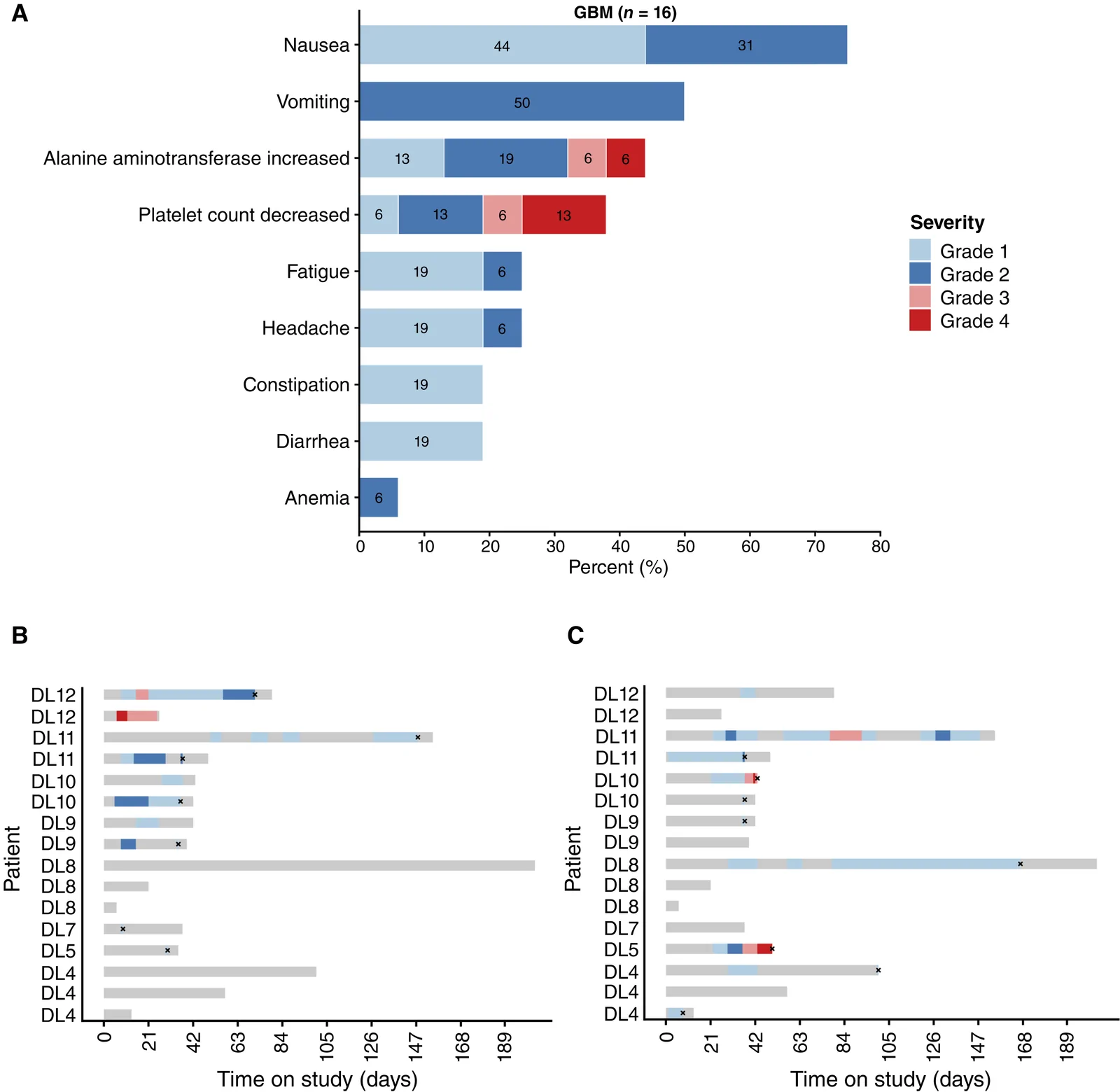
Patient safety profile for GBM. **A,** Common treatment-related AEs (≥10%) in patients with GBM. **B,** Swimmer plot of increased ALT based on reported lab values. **C,** Swimmer plot of decreased platelet count based on reported lab values. Lack of reported follow-up lab values is denoted by x.

We assessed whether the emergence of grade 3 to 4 AEs was related to LP-184 dose. Both patients with grade 3 or 4 ALT elevation received DL12, the highest dose and established DLT in the phase Ia clinical trial (Fig. 1B). No grade 2 or higher ALT elevation occurred below DL9. Conversely, there was no clear trend between platelet count decrease and LP-184 dose level (Fig. 1C).

We hypothesized that prior TMZ exposure may have increased the risk of liver toxicity and thrombocytopenia, given the known toxicity of TMZ. However, there was no clear relationship among the prior duration of TMZ, the number of prior TMZ therapies, and either TMZ-related ALT elevation or thrombocytopenia (Supplementary Fig. S1A–S1D).

### Preliminary responses

No radiographic responses were seen in patients with GBM per RANO 2.0 (Fig. 2A–C). Median progression-free survival was 1.3 months (range, 0.3–22 months). One patient (DL11) had stable disease for 4 months. A second patient (DL8) had radiographic progression on the first MRI response assessment but continued treatment for clinical benefit per his treating physician’s discretion. This patient’s pathology (confirmed at re-resection prior to study entry) revealed recurrent GBM, IDH wild type, WHO grade 4, MGMT promoter methylated. The patient received a total of 6 months of LP-184 before discontinuing treatment for prolonged low-grade neutropenia and thrombocytopenia complicated by frequent dose holds. To date, the patient continues to have clinically and radiographically stable disease for more than 22 months after enrollment (>17 months off treatment per communication with the treating physician) and has not received another line of therapy.

**Figure 2.**
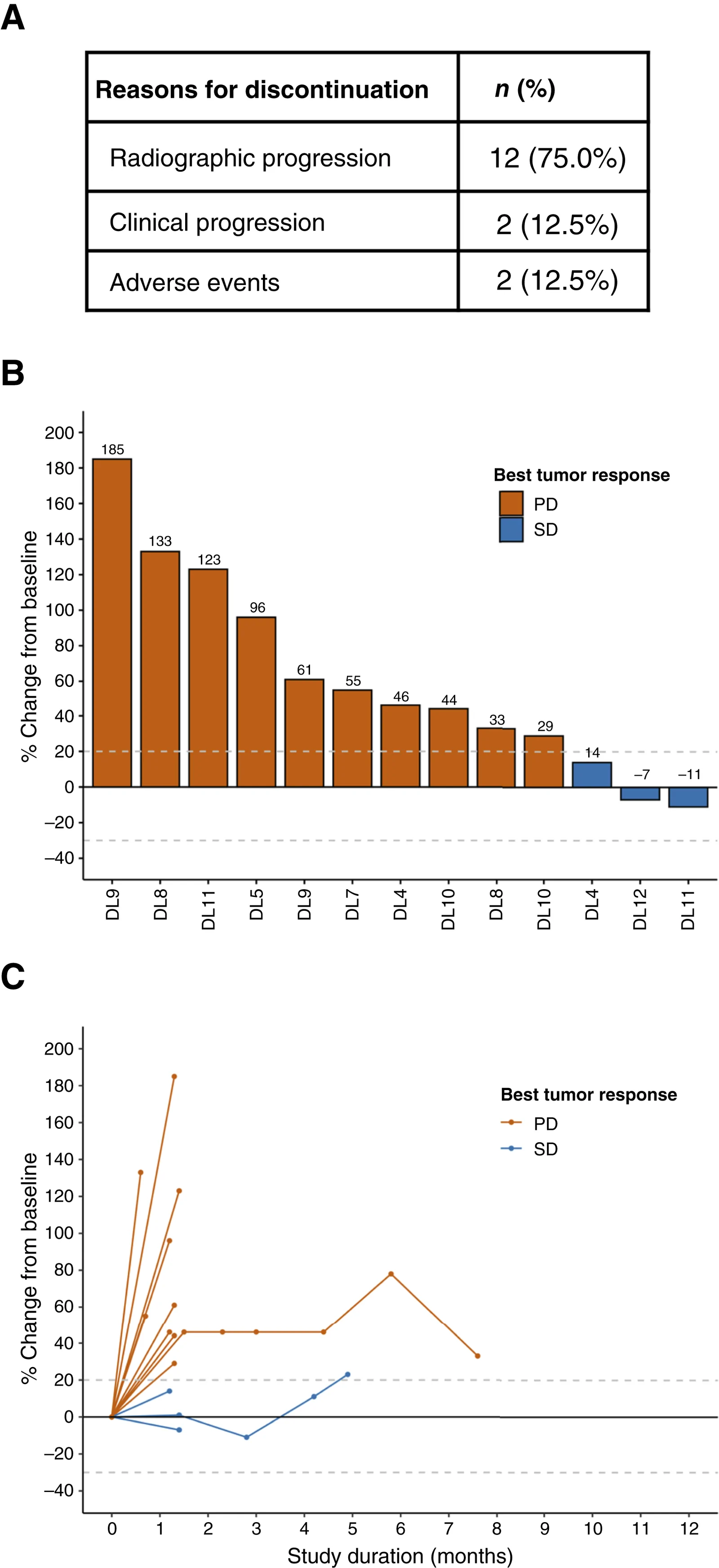
Response of patients with GBM per RANO 2.0. **A,** Reasons for treatment discontinuation. **B,** Waterfall plot with dose level (DL) indicated under each bar. **C,** Spider plot. PD, progressive disease; SD, stable disease.

### PTGR1 expression in clinical specimens

PTGR1 facilitates the conversion of the LP-184 prodrug to an active compound intracellularly. Because several attempts to quantify PTGR1 protein expression failed due to a lack of reliable antibodies, RNA expression was used as a surrogate. IC_50_s from 78 previously screened cancer cell lines (29, 30) were plotted against PTGR1 RNA expression levels obtained from CellMinerCDB RNA sequencing (RNA-seq) data (31). A therapeutic LP-184 concentration of 300 nmol/L was used as the sensitivity threshold, based on the average IC_50_ across solid tumor cell lines including human GBM isolates (288 nmol/L; ref. 20). This analysis identified a PTGR1 expression threshold of 4.5 log_2_(TPM + 1) as the cutoff associated with *in vitro* LP-184 sensitivity (IC_50_ < 300 nmol/L; Fig. 3A), where TPM stands for transcripts per million.

**Figure 3.**
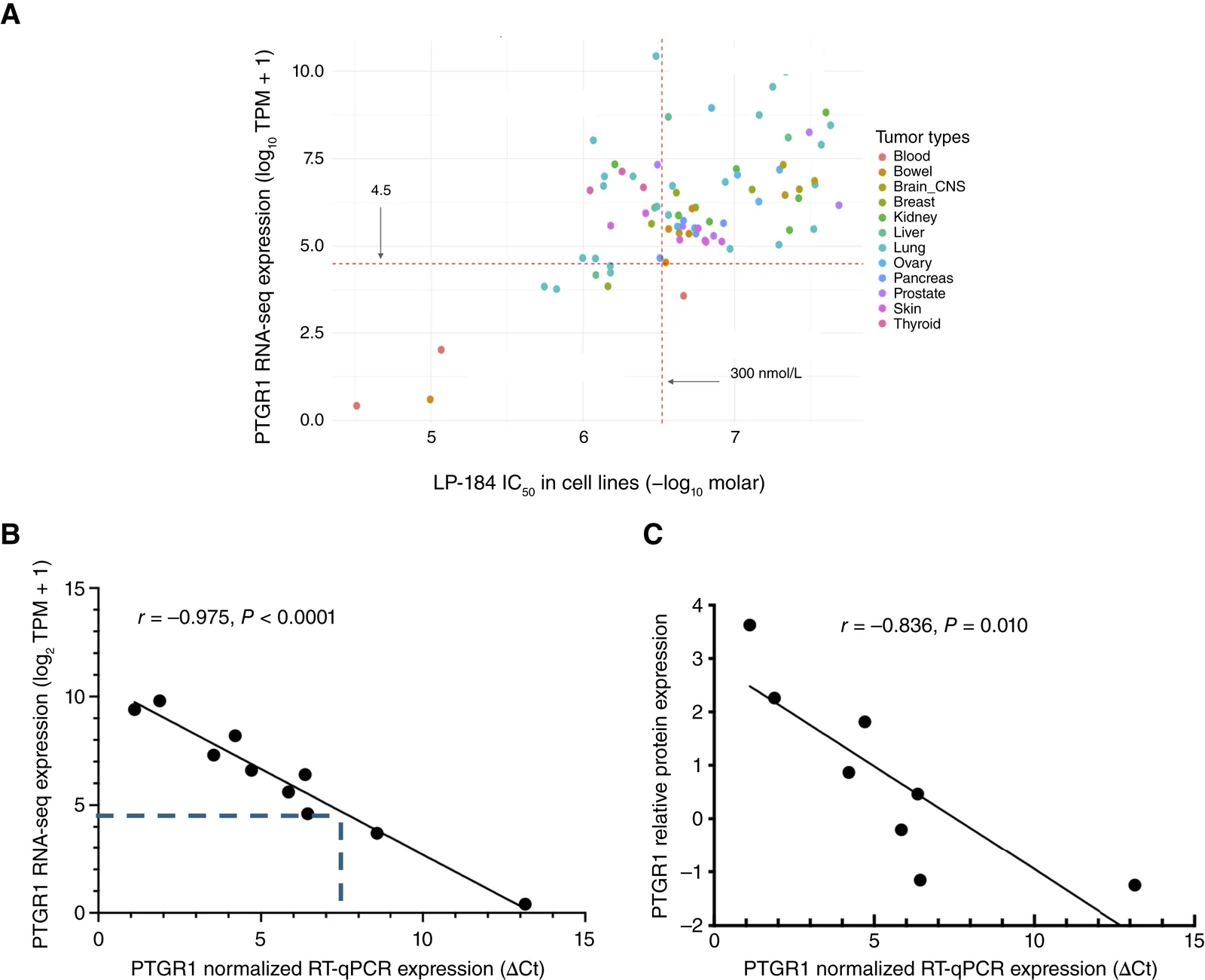
PTGR1 expression threshold measured by RT-qPCR. **A,** IC_50_s from 78 previously screened cancer cell lines were plotted against PTGR1 RNA expression levels obtained from CellMinerCDB RNA-seq data. PTGR1 expression level measured by RT-qPCR significantly correlated with the reported RNA-seq expression (**B**) and relative PTGR1 protein expression (**C**).

To enable clinical quantification of *PTGR1*, an RT-qPCR assay was developed (see “Patients and Methods”), with expression normalized against two housekeeping genes (*GAPDH* and *ACTB*). Validation against CellMinerCDB data confirmed linear correlations between RT-qPCR and both RNA-seq and protein expression across 10 cell lines. The 4.5 log_2_(TPM + 1) RNA-seq threshold corresponded to an RT-qPCR readout of approximately 7.5 ΔCT normalized PTGR1 (Fig. 3B and C).

Pretreatment tissue was available in 8 out of 16 subjects with GBM in the current phase Ia study, and 75% of these samples demonstrated PTGR1 expression above the defined threshold.

### Preclinical assessment and modeling of spironolactone-induced ERCC3 degradation kinetics

Spironolactone targets ERCC3 for ubiquitination and proteasomal degradation, and we have previously shown in multiple patient-derived GBM cell models that spironolactone sensitizes GBM cells to LP-184–induced cell death, resulting in a 3- to 6-fold lower IC_50_ and statistically significantly enhancing antitumor responses (20). With concerns that LP-184 administered at RDE (DL10) may not reach sufficient cytotoxic concentrations within the brain as a single agent, we sought to identify the translational potential of combining therapy with spironolactone. We evaluated how spironolactone dosing affects the timing and magnitude of ERCC3 protein degradation in an orthotopic GBM PDX model (Mayo39) to inform optimal spironolactone dosing for future clinical translation in combination with LP-184. Tumor ERCC3 levels were found to nadir at 8 hours (70% reduction) following a single dose of spironolactone (35 mg/kg intraperitoneally; Fig. 4A). We used the 8-hour postdose timing interval to examine the ERCC3 response to repetitive spironolactone dosing. Tumor ERCC3 levels were substantially lower (80% reduction) following three spironolactone doses (35 mg/kg intraperitoneally, every 24 hours) compared with either one dose or two consecutive doses (49%–57% reduction; Fig. 4B). Spironolactone had no effect on ERCC3 protein levels in the liver (Fig. 4C). Consistent with these results, the role of ERCC3 in DDR, and our prior published findings showing that spironolactone sensitizes GBM cells and orthotopic GBM PDXs (M1123 model) to LP-184, we found in orthotopic Mayo39 xenografts that administering spironolactone (35 mg/kg intraperitoneally, every 24 hours ×3) prior to a single dose of LP-184 (4 mg/kg intravenously) increased the tumor cell death response (cleaved caspase-3) by ∼2-fold compared with LP-184 alone (Fig. 4D).

**Figure 4.**
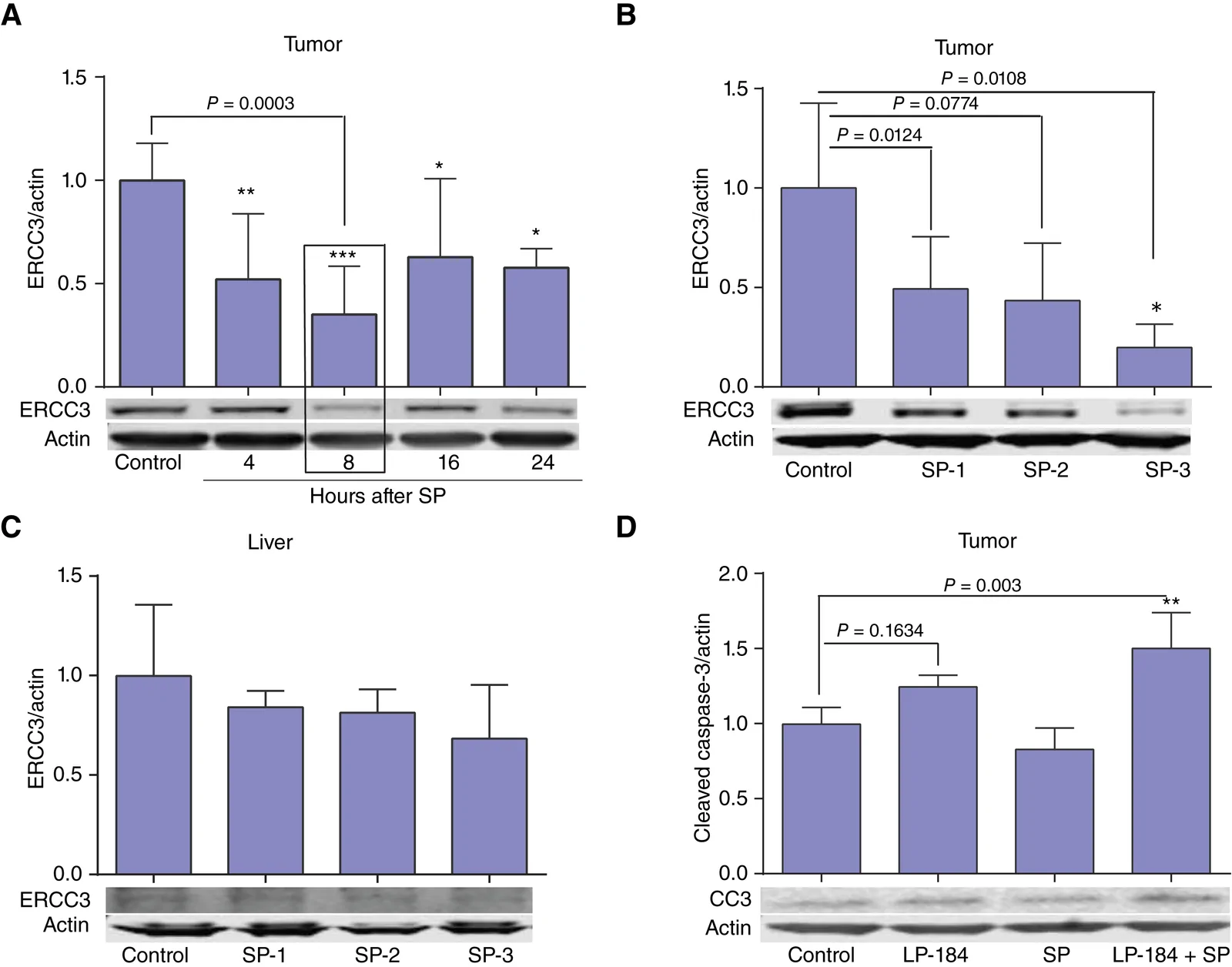
Spironolactone (SP) depletes ERCC3 in orthotopic GBM PDXs and enhances LP-184 tumor cytotoxicity. Mice (*N* = 4) with preestablished orthotopic Mayo39 xenografts were treated with SP (35 mg/kg, intraperitoneally) and/or LP-184 (4 mg/kg, intravenously) as described. Tissue-derived protein was analyzed by immunoblot, with representative blots shown. **A,** Kinetics of tumor ERCC3 depletion following a single dose of SP. **B,** Tumor ERCC3 levels quantified 8 hours following one, two, or three doses of SP (SP-1, SP-2, and SP-3, respectively) administered 24 hours apart. **C,** ERCC3 levels in livers obtained from mice treated as in **B**. **D,** Tumor caspase-3 activation (cleaved caspase-3) in response to a single dose of LP-184 alone, SP alone (three daily doses as in **B**), or LP-184 administered 8 hours following the last SP dose. Tumors were recovered for immunoblot analysis 48 hours after LP-184 infusion. *, *P* < 0.05; **, *P* < 0.005; ***, *P* < 0.0005.

### Pharmacokinetics

Plasma was collected at prespecified time points during and after study drug infusion from all patients participating in the phase Ia study. Plasma concentrations of LP-184 reached 500 nmol/L (C_max_) in the lower dose cohorts of LP-184 and were considerably higher (up to 900 nmol/L) at DL9 to DL12 (Fig. 5A and B; Supplementary Table S4). Notwithstanding, at all dose levels, plasma concentrations of LP-184 did not reach concentrations projected to achieve optimal on-target therapeutic concentrations in the brain based on our preclinical findings of 20% tumor penetration and 10% normal brain penetration (in mice) and human GBM cell IC_50_s (50–300 nmol/L; ref. 20).

**Figure 5.**
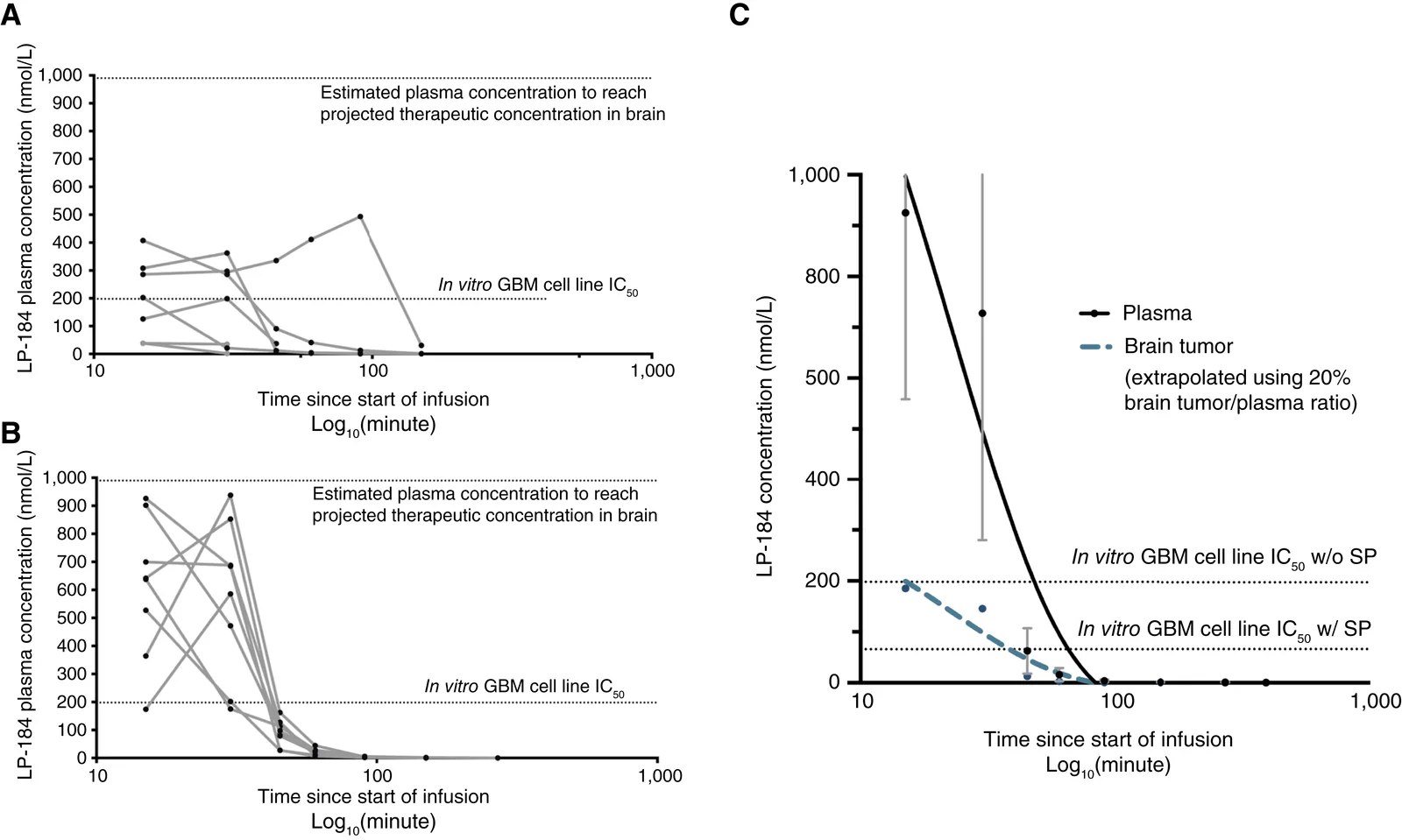
LP-184 PK (**A**) plasma concentration over time in patients with GBM, DL4–DL8 (*n* = 8), and (**B**) plasma concentration over time in patients with GBM, DL9–DL12 (*n* = 8). (**C**) Projected LP-184 therapeutic concentration in combination with spironolactone (SP). Nonlinear regression (on phase decay) was used to generate LP-184 plasma concentration–time curves based on data from 11 patients treated at dose level 10 (0.39 mg/kg). Brain tumor concentrations were extrapolated using a 20% brain tumor/plasma ratio. The IC_50_s with and without SP were derived from preclinical cell line data.

Using pharmacokinetic data from all patients treated with LP-184 at the RDE (DL10; *n* = 11, including two with GBM), we modeled projected LP-184 exposure in the presence of spironolactone (Fig. 5C). These simulations predict that coadministration of spironolactone with LP-184 at the RDE (DL10, C_max_ geometric mean = 839 nmol/L) will achieve intratumor LP-184 levels that exceed target cell IC_50_ by ∼2- to 3-fold.

## Discussion

Alkylating agents are the only chemotherapeutic class incorporated into the standard of care for GBM, and LP-184 exhibits properties that provide potential advantages compared with currently used alkylators. TMZ administered with radiation and following radiotherapy remains the standard for newly diagnosed GBM (1). TMZ alkylates guanine, generating O^6^-methylguanine adducts that promote mispairing during replication and are processed by the mismatch repair pathway (32). The nitrosourea lomustine (CCNU), routinely used at recurrence, induces interstrand cross-links that engage multiple DDR pathways, including Fanconi anemia and HR (33). The cytotoxic action of both TMZ and lomustine is mitigated, at least in part, by MGMT activity, which removes alkyl groups from guanine (3). In contrast, LP-184 preferentially alkylates adenine at the N3 position. The resultant DNA lesions are not susceptible to MGMT activity and are predominantly resolved by TC-NER rather than base excision repair or global NER pathways (34). Like lomustine, the cytotoxicity of LP-184 is enhanced in tumors with deficiencies in multiple DDR pathways, a vulnerability observed in EGFR-altered GBM based on *in silico* analysis (20).

The potency of LP-184 in selected cancers can be enhanced by a machine learning–guided drug repurposing strategy. These analyses *in silico* identified EGFR alterations and low *ERCC3* expression as predictors of LP-184 sensitivity (20). Spironolactone, a diuretic and known inhibitor of TC-NER, degrades *ERCC3* and sensitizes tumors to LP-184 (20). Here, we show that daily spironolactone administration for 3 consecutive days before LP-184 substantially reduces ERCC3 protein in orthotopic GBM PDXs and increases tumor cell death compared with LP-184 alone. Moreover, based on the RDE PK, the LP-184–spironolactone combination is projected to decrease the IC_50_ severalfold, thereby increasing chemosensitivity, a hypothesis to be confirmed in a planned future clinical trial.

Patients with GBM tolerated LP-184, supporting its potential for combination strategies and further study in patients with GBM. LP-184 toxicity was broadly consistent with that observed in other solid cancers. No clear association was observed between dose level and nausea or thrombocytopenia; in contrast, serious ALT elevation increased with dose. We hypothesized that prior TMZ exposure might predispose patients to thrombocytopenia, given its known myelosuppressive effects, but did not detect a significant association between prior TMZ and thrombocytopenia risk. This lack of association may reflect limited sample size or the relative uniformity of prior TMZ exposure in the study GBM cohort. Although no objective responses to LP-184 monotherapy were observed, one participant with the first recurrence of GBM had early radiographic progression followed by stable disease for a total of eight cycles (6 months) of LP-184. The patient was taken off treatment for recurrent, low-grade myelosuppression resulting in frequent dose holds but currently remains progression- and treatment-free more than 17 months after stopping LP-184 treatment.

LP-184 is a novel prodrug of the acylfulvene class that is well tolerated, and an identified RDE in solid cancers, including GBM, was determined. Its distinct alkylation site and activity independent of MGMT status support further development in GBM and other malignancies characterized by deficiencies in DDR pathways. Nevertheless, its limited CNS penetration suggests that monotherapy is unlikely to achieve clinically meaningful activity in patients with brain cancers. Combination therapy with spironolactone, a BBB-permeable inhibitor of NER, shows promising preclinical efficacy. Here, we have identified the systemic concentration of LP-184 in human patients and the RDE for future clinical trials. Moreover, we have established a clinically translatable strategy for dosing the ERCC3 degrader, spironolactone, in combination with LP-184 in preparation for a phase Ib/IIa study in patients with the first recurrence of GBM following standard-of-care TMZ-based therapy (NCT07431216).

## Supplementary Material

Supplementary Figure 1 Association between prior treatments and lab values.

Supplementary Table 1 Representativeness of trial and background on glioblastoma

Supplementary Table 2 Molecular features of study participants with glioblastoma

Supplementary Table 3 Patient enrollment per dose level

Supplementary Table 4 LP-184 non-compartmental pharmacokinetic parameters

## Data Availability

The data generated in this study are available within the article and its supplementary data files. Sample-level data from preclinical studies are available upon reasonable requests to the corresponding author.

## References

[1] Stupp R , Mason WP, van den Bent MJ, Weller M, Fisher B, Taphoorn MJ, . Radiotherapy plus concomitant and adjuvant temozolomide for glioblastoma. N Engl J Med 2005;352:987–96. 10.1056/NEJMoa043330. 15758009

[2] Price M , Ballard C, Benedetti J, Neff C, Cioffi G, Waite KA, . CBTRUS statistical report: primary brain and other central nervous system tumors diagnosed in the United States in 2017–2021. Neuro Oncol 2024;26(Supplement_6):vi1–85. 10.1093/neuonc/noae145. PMC11456825 39371035

[3] Hegi ME , Diserens AC, Gorlia T, Hamou MF, de Tribolet N, Weller M, . MGMT gene silencing and benefit from temozolomide in glioblastoma. N Engl J Med 2005;352:997–1003. 10.1056/NEJMoa043331. 15758010

[4] Wen PY , Weller M, Lee EQ, Touat M, Khasraw M, Rahman R, . Glioblastoma in adults: a Society for Neuro-Oncology (SNO) and European Society of Neuro-Oncology (EANO) consensus review on current management and future directions. Neuro Oncol 2025;27:2751–88. 10.1093/neuonc/noaf177. PMC12908567 40827022

[5] Neftel C , Laffy J, Filbin MG, Hara T, Shore ME, Rahme GJ, . An integrative model of cellular states, plasticity, and genetics for glioblastoma. Cell 2019;178:835–49.e21. 10.1016/j.cell.2019.06.024. PMC6703186 31327527

[6] Verhaak RG , Hoadley KA, Purdom E, Wang V, Qi Y, Wilkerson MD, . Integrated genomic analysis identifies clinically relevant subtypes of glioblastoma characterized by abnormalities in PDGFRA, IDH1, EGFR, and NF1. Cancer Cell 2010;17:98–110. 10.1016/j.ccr.2009.12.020. PMC2818769 20129251

[7] Pardridge WM . Blood-brain barrier delivery. Drug Discov Today 2007;12:54–61. 10.1016/j.drudis.2006.10.013. 17198973

[8] Sarkaria JN , Hu LS, Parney IF, Pafundi DH, Brinkmann DH, Laack NN, . Is the blood-brain barrier really disrupted in all glioblastomas? A critical assessment of existing clinical data. Neuro Oncol 2018;20:184–91. 10.1093/neuonc/nox175. PMC5777482 29016900

[9] Arvanitis CD , Ferraro GB, Jain RK. The blood-brain barrier and blood-tumour barrier in brain tumours and metastases. Nat Rev Cancer 2020;20:26–41. 10.1038/s41568-019-0205-x. PMC8246629 31601988

[10] Grossman SA , Barone A, Strowd RE, Rais R, Duke E, Wearne E, . Evaluating “brain permeability”: a critical issue for the development of therapeutic agents for primary and metastatic brain tumors. Neuro Oncol 2026;28:1599–611. 10.1093/neuonc/noag051. PMC13338342 41818680

[11] Yu X , Erzinger MM, Pietsch KE, Cervoni-Curet FN, Whang J, Niederhuber J, . Up-regulation of human prostaglandin reductase 1 improves the efficacy of hydroxymethylacylfulvene, an antitumor chemotherapeutic agent. J Pharmacol Exp Ther 2012;343:426–33. 10.1124/jpet.112.195768. PMC3477217 22895897

[12] Kathad U , Kulkarni A, McDermott JR, Wegner J, Carr P, Biyani N, . A machine learning-based gene signature of response to the novel alkylating agent LP-184 distinguishes its potential tumor indications. BMC Bioinformatics 2021;22:102. 10.1186/s12859-021-04040-8. PMC7923321 33653269

[13] Xue L , Zhu Z, Wang Z, Li H, Zhang P, Wang Z, . Knockdown of prostaglandin reductase 1 (PTGR1) suppresses prostate cancer cell proliferation by inducing cell cycle arrest and apoptosis. Biosci Trends 2016;10:133–9. 10.5582/bst.2016.01045. 27150108

[14] Wang X , Yin G, Zhang W, Song K, Zhang L, Guo Z. Prostaglandin reductase 1 as a potential therapeutic target for cancer therapy. Front Pharmacol 2021;12:717730. 10.3389/fphar.2021.717730. PMC8377670 34421612

[15] Koeppel F , Poindessous V, Lazar V, Raymond E, Sarasin A, Larsen AK. Irofulven cytotoxicity depends on transcription-coupled nucleotide excision repair and is correlated with XPG expression in solid tumor cells. Clin Cancer Res 2004;10:5604–13. 10.1158/1078-0432.CCR-04-0442. 15328203

[16] Wang J , Wiltshire T, Wang Y, Mikell C, Burks J, Cunningham C, . ATM-dependent CHK2 activation induced by anticancer agent, irofulven. J Biol Chem 2004;279:39584–92. 10.1074/jbc.M400015200. 15269203

[17] Wang Y , Wiltshire T, Senft J, Wenger SL, Reed E, Wang W. Fanconi anemia D2 protein confers chemoresistance in response to the anticancer agent, irofulven. Mol Cancer Ther 2006;5:3153–61. 10.1158/1535-7163.MCT-06-0427. 17172419

[18] Restifo D , McDermott JR, Cvetkovic D, Dos Santos T, Ogier C, Surumbayeva A, . Conditional dependency of LP-184 on prostaglandin reductase 1 is synthetic lethal in pancreatic cancers with DNA damage repair deficiencies. Mol Cancer Ther 2023;22:1182–90. 10.1158/1535-7163.MCT-22-0818. PMC10592171 37552607

[19] Kulkarni A , Zhou J, Biyani N, Kathad U, Banerjee PP, Srivastava S, . LP-184, a novel acylfulvene molecule, exhibits anticancer activity against diverse solid tumors with homologous recombination deficiency. Cancer Res Commun 2024;4:1199–210. 10.1158/2767-9764.CRC-23-0554. PMC11072798 38630886

[20] Lal B , Kulkarni A, McDermott J, Rais R, Alt J, Wu Y, . Preclinical efficacy of LP-184, a tumor site activated synthetic lethal therapeutic, in glioblastoma. Clin Cancer Res 2023;29:4209–18. 10.1158/1078-0432.CCR-23-0673. 37494541

[21] Shahar OD , Kalousi A, Eini L, Fisher B, Weiss A, Darr J, . A high-throughput chemical screen with FDA approved drugs reveals that the antihypertensive drug Spironolactone impairs cancer cell survival by inhibiting homology directed repair. Nucleic Acids Res 2014;42:5689–701. 10.1093/nar/gku217. PMC4027216 24682826

[22] Xu D , Cao Q, Wang L, Wang J, Xu B, Attwood K, . A preclinical study to repurpose spironolactone for enhancing chemotherapy response in bladder cancer. Mol Cancer Ther 2022;21:786–98. 10.1158/1535-7163.MCT-21-0613. PMC9081199 35247903

[23] Alekseev S , Ayadi M, Brino L, Egly JM, Larsen AK, Coin F. A small molecule screen identifies an inhibitor of DNA repair inducing the degradation of TFIIH and the chemosensitization of tumor cells to platinum. Chem Biol 2014;21:398–407. 10.1016/j.chembiol.2013.12.014. 24508195

[24] Börcsök J , Sztupinszki Z, Bekele R, Gao SP, Diossy M, Samant AS, . Identification of a synthetic lethal relationship between nucleotide excision repair deficiency and irofulven sensitivity in urothelial cancer. Clin Cancer Res 2021;27:2011–22. 10.1158/1078-0432.CCR-20-3316. PMC8026514 33208343

[25] Gabbard RD , Hoopes RR, Kemp MG. Spironolactone and XPB: an old drug with a new molecular target. Biomolecules 2020;10:756. 10.3390/biom10050756. PMC7277409 32414008

[26] Lopez-Bertoni H , Johnson A, Rui Y, Lal B, Sall S, Malloy M, . Sox2 induces glioblastoma cell stemness and tumor propagation by repressing TET2 and deregulating 5hmC and 5mC DNA modifications. Signal Transduct Target Ther 2022;7:37. 10.1038/s41392-021-00857-0. PMC8826438 35136034

[27] Mahadevan D , Parekh J, Beck JT, McKean WB Jr, Schreck KC, Olszanski AJ, . A first-in-human phase 1a study of LP-184, a tumor-site activated novel alkylating agent, in patients with advanced solid tumors. Invest New Drugs 2026 Jul 7 [Epub ahead of print]. 10.1007/s10637-026-01626-y. 42410111

[28] Vaubel RA , Tian S, Remonde D, Schroeder MA, Mladek AC, Kitange GJ, . Genomic and phenotypic characterization of a broad panel of patient-derived xenografts reflects the diversity of glioblastoma. Clin Cancer Res 2020;26:1094–104. 10.1158/1078-0432.CCR-19-0909. PMC7056576 31852831

[29] McDermott J , Sturtevant D, Kathad U, Varma S, Zhou J, Kulkarni A, . Artificial intelligence platform, RADR®, aids in the discovery of DNA damaging agent for the ultra-rare cancer Atypical Teratoid Rhabdoid Tumors. Front Drug Discov 2022;2:1033395. 10.3389/fddsv.2022.1033395.

[30] Kulkarni A , McDermott JR, Kathad U, Modali R, Richard JP, Sharma P, . The acylfulvene alkylating agent, LP-184, retains nanomolar potency in non-small cell lung cancer carrying otherwise therapy-refractory mutations. Oncotarget 2021;12:791–806. 10.18632/oncotarget.27943. PMC8057270 33889302

[31] Luna A , Elloumi F, Varma S, Wang Y, Rajapakse VN, Aladjem MI, . CellMiner Cross-Database (CellMinerCDB) version 1.2: exploration of patient-derived cancer cell line pharmacogenomics. Nucleic Acids Res 2021;49:D1083–93. 10.1093/nar/gkaa968. PMC7779001 33196823

[32] Schreck KC , Grossman SA. Role of temozolomide in the treatment of cancers involving the central nervous system. Oncology (Williston Park) 2018;32:555–60, 569. 30474103

[33] Dolan ME , Moschel RC, Pegg AE. Depletion of mammalian O6-alkylguanine-DNA alkyltransferase activity by O6-benzylguanine provides a means to evaluate the role of this protein in protection against carcinogenic and therapeutic alkylating agents. Proc Natl Acad Sci U S A 1990;87:5368–72. 10.1073/pnas.87.14.5368. PMC54325 2164681

[34] Staake MD , Kashinatham A, McMorris TC, Estes LA, Kelner MJ. Hydroxyurea derivatives of irofulven with improved antitumor efficacy. Bioorg Med Chem Lett 2016;26:1836–8. 10.1016/j.bmcl.2016.02.028. 26922141

