# Supplementary material for: Phase Ia Evaluation of LP-184 in Recurrent Glioblastoma: Safety, Pharmacokinetics, and Translational Optimization of CNS Exposure
Source: Cancer Res Commun. 2026 Oct 27;6(10):2519–28. doi: 10.1158/2767-9764.CRC-26-0518 (PMC13711432; doi:10.1158/2767-9764.CRC-26-0518)
Supplement: Supplementary file 1 [file crc-26-0518_supplementary_figure_1_suppsf1.docx]

**Supplemental Fig. 1.** Association between number of prior treatments or prior TMZ duration with increased ALT (A, B) and decreased PLT (C, D). TMZ, temozolomide; ALT, alanine aminotransferase; PLT, platelet count.
