# Supplementary material for: Phase Ia Evaluation of LP-184 in Recurrent Glioblastoma: Safety, Pharmacokinetics, and Translational Optimization of CNS Exposure
Source: Cancer Res Commun. 2026 Oct 27;6(10):2519–28. doi: 10.1158/2767-9764.CRC-26-0518 (PMC13711432; doi:10.1158/2767-9764.CRC-26-0518)
Supplement: Supplementary file 2 [file crc-26-0518_supplementary_table_1_suppst1.docx]

**Supplemental Table 1.** Representativeness of trial and background on glioblastoma.

| Cancer type | Glioblastoma (GBM |
| --- | --- |
| Sex | Glioblastoma occurs more frequently in males than females. In US registry data from 2018–2022, the age-adjusted incidence rates were approximately 4.07 per 100,000 males and 2.60 per 100,000 females. Thus, a study containing approximately 60% males and 40% females may broadly reflect the sex distribution of the underlying US glioblastoma population. |
| Age | The median age at diagnosis in the United States is approximately 66 years, with incidence increasing sharply after age 40 and peaking in the early 70s. Older adults have historically been underrepresented in glioblastoma trials: an analysis of 118 US interventional trials conducted from 1994–2019 found a participant median age of 54 years, approximately 10 years younger than the corresponding population median. |
| Race/ethnicity | US age-adjusted incidence rates per 100,000 during 2018–2022 were approximately 3.73 among non-Hispanic White persons, 2.64 among Hispanic persons, 2.37 among non-Hispanic American Indian/Alaska Native persons, 1.91 among non-Hispanic Black persons, and 1.72 among non-Hispanic Asian or Pacific Islander persons. These categories are descriptive registry classifications and should not be interpreted as evidence of innate biological differences. |
| Geography | The average annual US glioblastoma incidence is approximately 3.28 per 100,000, with an estimated 13,930 new US diagnoses in 2025. |
| Other Considerations | Representativeness may be affected by eligibility requirements involving Karnofsky Performance Status or ECOG status, cognitive capacity, corticosteroid dose, organ function, comorbidities, prior treatment, tumor location, availability of tumor tissue, and ability to undergo surgery. Important disease characteristics include newly diagnosed versus recurrent disease, MGMT promoter-methylation status, extent of resection or biopsy only, measurable disease requirements, number of prior recurrences, and prior radiotherapy or systemic therapy. |
| Overall representativeness of this study | The age distribution of our study is similar to the average age distribution in other studies in glioblastoma and approximately 10 years younger than the average age of diagnosis with glioblastoma. The sex distribution is as expected, and given the national enrollment of this trial we have included geographic representation to the best of our ability. |
