# Supplementary material for: Phase Ia Evaluation of LP-184 in Recurrent Glioblastoma: Safety, Pharmacokinetics, and Translational Optimization of CNS Exposure
Source: Cancer Res Commun. 2026 Oct 27;6(10):2519–28. doi: 10.1158/2767-9764.CRC-26-0518 (PMC13711432; doi:10.1158/2767-9764.CRC-26-0518)
Supplement: Supplementary file 3 [file crc-26-0518_supplementary_table_2_suppst2.docx]

**Supplemental Table 2.** Molecular features of study participants with glioblastoma. Gene alterations were retrieved from enrollment packages. PTGR1 RNA expression was assessed by RT-qPCR using archival tumor tissue as described in the method section.

| **Subject** | **IDH** | **pMGMT methylation** | **EGFR alteration** | **PTEN mut** | **PI3K mut** | **pTERT mut** | **ATRX mut** | **CDKN2 A/B deletion** | **PDGFR amplification** | **PTGR1 RNA Expression (ΔCt)** |
| --- | --- | --- | --- | --- | --- | --- | --- | --- | --- | --- |
| 1 | WT | Methylated | No | No | No | c.146C>T | No | CDKN2B del | No | NA |
| 2 | WT | Unmethylated | EGFR amp | Not Reported | Not Reported | c.146C>T | Not Reported | CDKN2A/2B codeletion | Not Reported | NA |
| 3 | WT | Unknown | No | p.R173C | No | c.146C>T | No | Not Reported | No | 5.97 |
| 4 | WT | Methylated | EGFRviii EGFR amp | Not Reported | Not Reported | Not Reported | Not Reported | Not Reported | Not Reported | NA |
| 5 | WT | Unmethylated | EGFRviii EGFR amp | No | No | c.146C>T | No | CDKN2A/2B codeletion | No | 7.42 |
| 6 | WT | Unmethylated | EGFRviii EGFR amp | No | p.G376R | c.146C>T | No | Not Reported | No | NA |
| 7 | WT | Unmethylated | No | p.R335P | No | c.146C>T | No | Not Reported | No | NA |
| 8 | WT | Unmethylated | EGFR amp | Not Reported | Not Reported | Not Reported | Not Reported | Not Reported | Not Reported | NA |
| 9 | WT | Methylated | EGFR amp | No | No | Detected | No | No | No | 7.11 |
| 10 | WT | Methylated | EGFRviii EGFR amp | p.K128N | Not Reported | c.146C>T | Not Reported | Not Reported | Not Reported | NA |
| 11 | WT | Methylated | EGFRviii EGFR amp | No | p.P104R | c.146C>T | No | CDKN2A/2B codeletion | No | 7.09 |
| 12 | WT | Methylated | Not Reported | Not Reported | Not Reported | Not Reported | Not Reported | Not Reported | Not Reported | 9.18 |
| 13 | WT | Unmethylated | Not Reported | Not Reported | Not Reported | Not Reported | Not Reported | Not Reported | Not Reported | 7.32 |
| 14 | WT | Unmethylated | Not Reported | Not Reported | Not Reported | Not Reported | Not Reported | Not Reported | Not Reported | 7.88 |
| 15 | WT | Unmethylated | Not Reported | Not Reported | Not Reported | Not Reported | Mutant | Not Reported | Not Reported | 5.71 |
| 16 | WT | Not Reported | No | No | No | c.146C>T | Yes | CDKN2B del | Amplified | NA |
