# Supplementary material for: Phase Ia Evaluation of LP-184 in Recurrent Glioblastoma: Safety, Pharmacokinetics, and Translational Optimization of CNS Exposure
Source: Cancer Res Commun. 2026 Oct 27;6(10):2519–28. doi: 10.1158/2767-9764.CRC-26-0518 (PMC13711432; doi:10.1158/2767-9764.CRC-26-0518)
Supplement: Supplementary file 4 [file crc-26-0518_supplementary_table_3_suppst3.docx]

**Supplemental Table 3.** Patient enrollment per dose level.

| **Treated per Dose Level** | **n (%)**  **Total N=16** |
| --- | --- |
| DL01 (0.01 mg/kg) | 0 |
| DL02 (0.02 mg/kg) | 0 |
| DL03 (0.04 mg/kg) | 0 |
| DL04 (0.07 mg/kg) | 3 (19%) |
| DL05 (0.11 mg/kg) | 1 (6%) |
| DL06 (0.15 mg/kg) | 0 |
| DL07 (0.20 mg/kg) | 1 (6%) |
| DL08 (0.25 mg/kg) | 3 (19%) |
| DL09 (0.31 mg/kg) | 2 (13%) |
| DL10 (0.39 mg/kg) | 2 (13%) |
| DL11 (0.49 mg/kg) | 2 (13%) |
| DL12 (0.61 mg/kg) | 2 (13%) |
