# Supplementary material for: Phase Ia Evaluation of LP-184 in Recurrent Glioblastoma: Safety, Pharmacokinetics, and Translational Optimization of CNS Exposure
Source: Cancer Res Commun. 2026 Oct 27;6(10):2519–28. doi: 10.1158/2767-9764.CRC-26-0518 (PMC13711432; doi:10.1158/2767-9764.CRC-26-0518)
Supplement: Supplementary file 5 [file crc-26-0518_supplementary_table_4_suppst4.docx]

**Supplemental Table 4.** LP-184 non-compartmental pharmacokinetic parameters derived from nominal plasma concentration-time data in glioblastoma patients.

|  | **DL04 (0.07 mg/kg)** | **DL05 (0.11 mg/kg)** | **DL7 (0.20 mg/kg)** | **DL8 (0.25 mg/kg)** | **DL9 (0.31 mg/kg)** | **DL10 (0.39 mg/kg)** | **DL11 (0.49 mg/kg)** | **DL12 (0.61 mg/kg)** |
| --- | --- | --- | --- | --- | --- | --- | --- | --- |
| **N** | 3 | 1 | 1 | 3 | 2 | 2 | 2 | 2 |
| **C_max_ (nM)** | 179 (3.8) | 199 (1.0) | 203 (1.0) | 179 (3.8) | 690 (1.5) | 738 (1.2) | 805 (1.2) | 741 (1.4) |
| **Auc_last_ (nM*hr)** | 99 (6.7) | 80 (1.0) | 54 (1.0) | 63 (6.1) | 255 (1.6) | 292 (1.4) | 365 (1.0) | 265 (1.5) |
| **T_max_ (min)** | 45 (15-90) | 30 | 15 | 20 (15-30) | 15 (15-15) | 23 (15-30) | 15 (15-15) | 30 (30-30) |
| **Half life (min)** | ND | ND | 31 | 15 (12-18) | 22 (19-24) | 37 (17-56) | 18 (12-23) | 18 (14-21) |

C_max_, and AUC_last_, are reported as geometric mean (geometric standard deviation factor); Half-life (apparent elimination half-life) and T_max_ are reported as mean (minimum-maximum); C_max_, maximum plasma concentration; AUC_last_, area under the curve to the last quantifiable plasma concentration; T_max_, time required to reach the maximum plasma concentration;  ND, not determined
